# Supplementary material for: Hierarchical regulation of functionally antagonistic neuropeptides expressed in a single neuron pair
Source: Nat Commun. 2024 Nov 3;15:9504. doi: 10.1038/s41467-024-53899-7 (PMC11532408; doi:10.1038/s41467-024-53899-7)
Supplement: Supplementary file 1 — Supplementary Information [file 41467_2024_53899_MOESM1_ESM.pdf]

# Supplementary Information

## Hierarchical regulation of functionally antagonistic neuropeptides expressed in a single neuron pair

Aoki et al.

Supplementary Fig. 1 (related to Fig. 1)

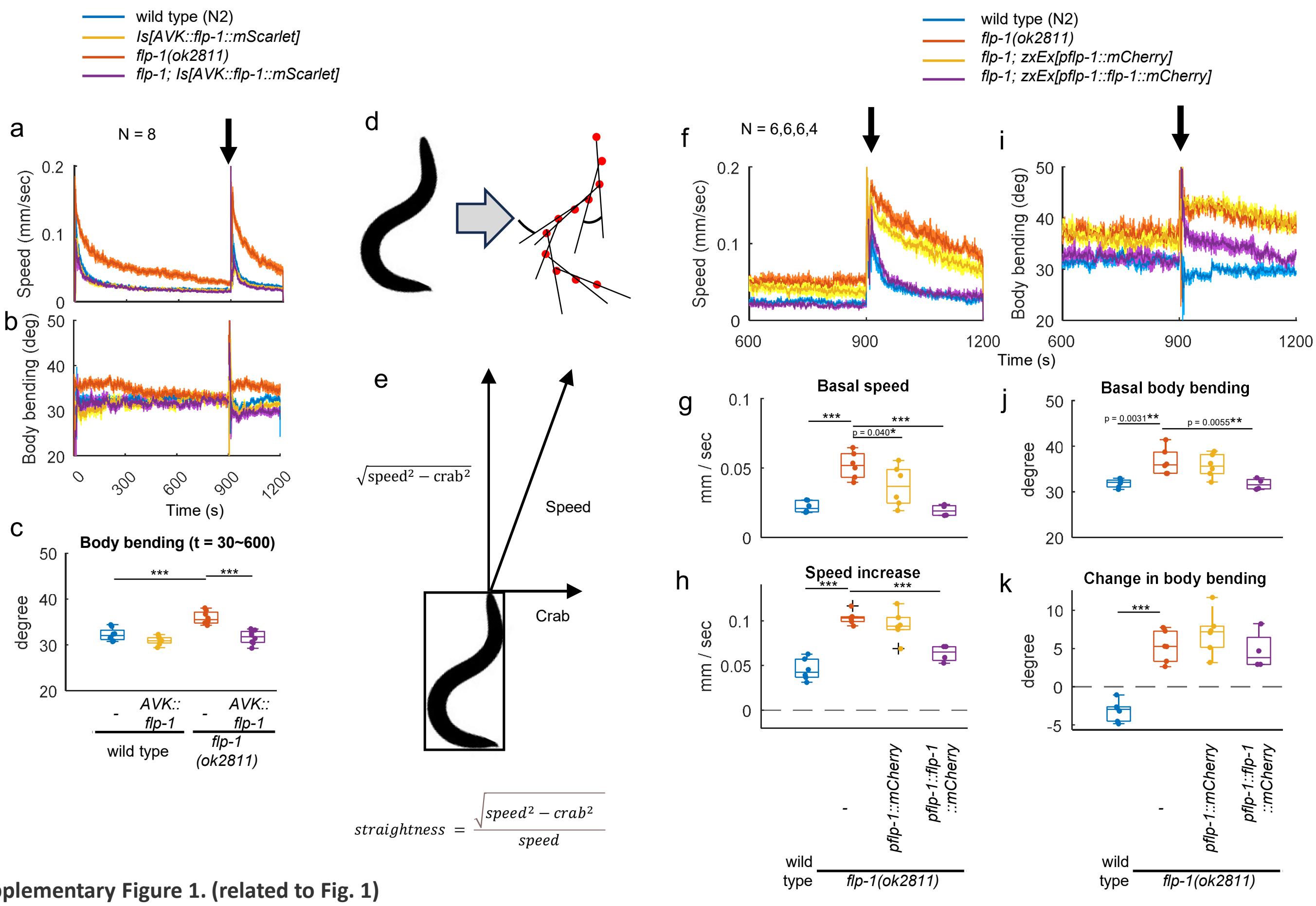


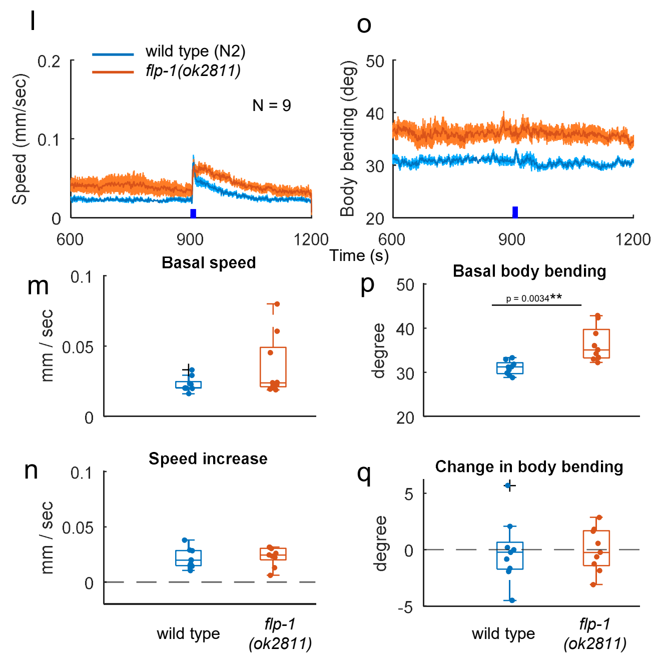


**Supplementary Figure 1. (related to Fig. 1)**

**a-c.** Representation of the whole recording duration (from 1 to 1200 sec, ii and iv) of data shown in Fig. 1b. Mean body bending angles from t = 30 to 600 were plotted in iii. Tukey test was performed. ***p < 0.001.

**d.** The MWT software saves an eleven-point spine along the center of every tracked animal and extracts 'Body bending'[1].

**e.** 'Straightness' is defined as the ratio of the vectorial speed component parallel to the orientation of the main body axis, relative to the overall speed within 1 second (see Methods).

**f-k.** 60 animals of indicated genotype were transferred to a NGM plate with food one day before the experiment. Animals' locomotion was tracked with MWT. NGM plates were tapped three times at 1 Hz, 900 seconds after recording was started. Basal speed (g) and body bending (j) indicate mean values of each from t = 840 to 899. 'Speed increase' (h) and 'Change in body bending' (k) indicate mean values from t = 911 to 960 subtracted by basal values. Dunnett test was performed against *flp-1* mutants. ***p < 0.001. Approximately 20-50 animals were involved in each recording.

**l-q.** Wild type or *flp-1(ok2811)* animals on NGM plates with bacterial food were subjected to behavioral analysis with MWT, while blue light was illuminated for 2 seconds at 900 seconds at 1.3 mW/mm$^2$. Basal speed (m) and body bending (p) indicate mean values of each from t = 840 to 899. 'Speed increase' (n) and 'Change in body bending' (q) indicate mean values from t = 901 to 960 subtracted by basal values. Two-tailed Welch test was performed. ***p < 0.001. Approximately 20-50 animals were involved in each recording.

In timeseries plots, data are presented as mean values +/- SEM. In boxplots, the boxes extend from the Q1 to Q3, with the band inside the boxes representing the median. The whiskers extend to the smallest and largest values within 1.5 times the IQR. Source data are provided in 'Source Data1' file.

Supplementary Fig. 2 (related to Fig. 1)

a. *nlp-49* locus

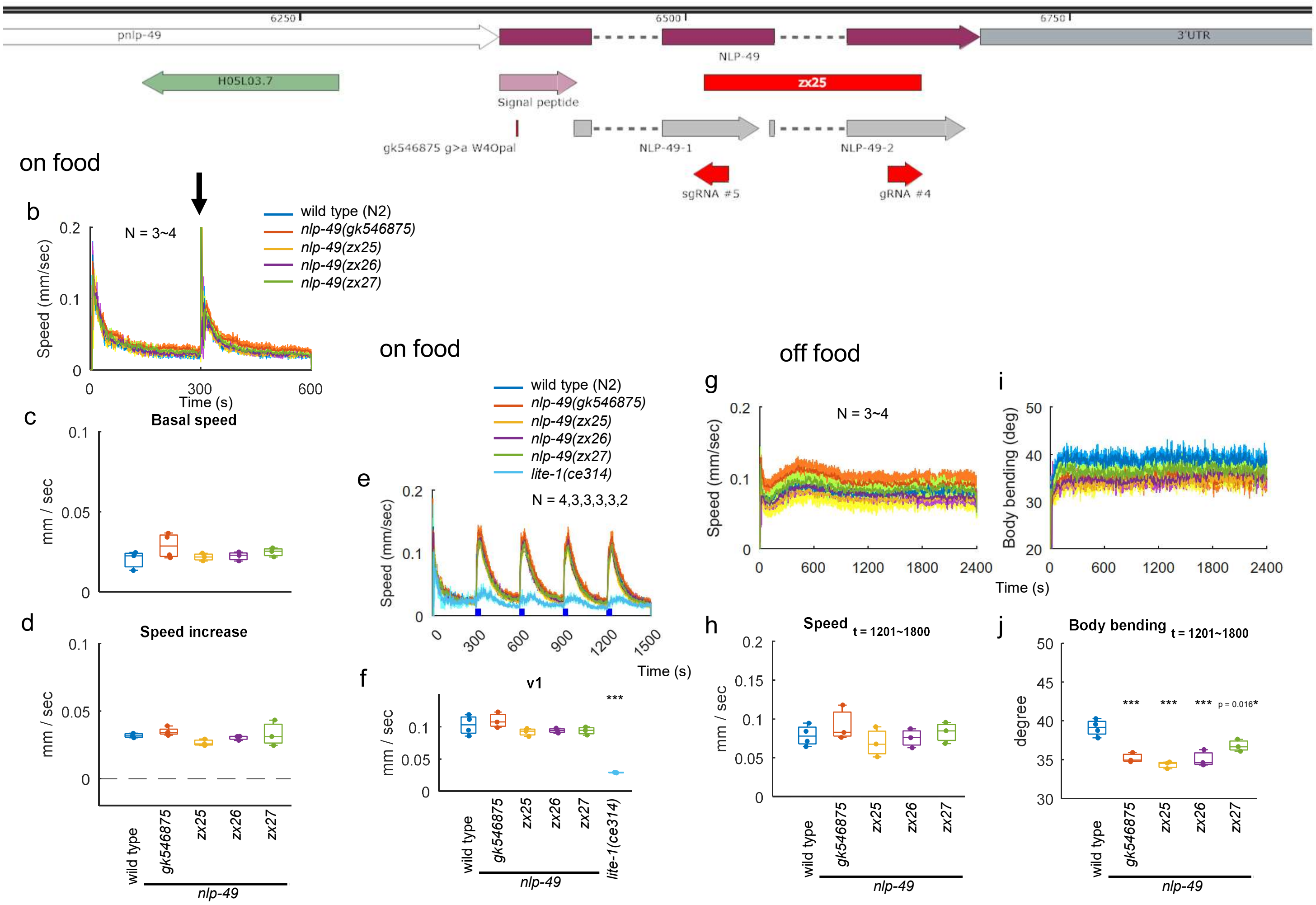


**Supplementary Figure 2. (related to Fig. 1)**

As the stop codon generated in the *nlp-49(gk546875)* allele could potentially be read through[2–4], we generated distinct *nlp-49* deletion alleles and characterized their locomotion (**Supplementary Fig. 2a**). Both *nlp-49* deletion mutants and the *gk546875* allele showed no remarkable defects in locomotion on food and exhibited normal speed increase upon tapping or in response to blue light illumination (**Supplementary Fig. 2b-f**). However, *nlp-49* mutants displayed decreased body bending angles during locomotion off food, consistent with a previous report for *nlp-49(gk546875)* mutants[5] (**Supplementary Fig. 2g-j**). These results confirm that *nlp-49(gk546875)* is indeed a nonsense allele.

**a.** *nlp-49* genomic locus. Positions of mutations were indicated.

**b-d.** Animals of indicated genotypes on NGM plates with bacterial food were subjected to behavioral analysis with MWT, while mechanical stimuli were given by tapping plates three times at 1 Hz, 300 seconds after recording started, as indicated by an arrow. Locomotion speed along time (b), average basal locomotion speed before tapping (c, t = 180 ~ 299, $v_0$) and speed increase after tapping (d, average speed (t = 300 ~ 420) – $v_0$) were plotted. Approximately 20-100 animals were involved in each recording.

**e-f.** Animals of indicated genotypes on NGM plates with bacterial food were subjected to behavioral analysis with MWT, while blue light was illuminated for 30 seconds at 300, 600, 900 and 1200 seconds. Locomotion speed along time (e) and average locomotion speed during the first blue light illumination (f, t = 300 ~ 330, $v_1$) were plotted. Dunnett test was performed against wild type. ***$p < 0.001$. Approximately 20-160 animals were involved in each recording.

**g-j.** Animals of indicated genotypes were washed and transferred to NGM plates without bacterial food, and their locomotion was analyzed with MWT. Locomotion speed (g) and body bending angle (i) along time, and average locomotion speed (h) and body bending (j) (t = 1201 ~ 1800) were plotted. Dunnett test was performed against wild type. ***$p < 0.001$. Approximately 20-100 animals were involved in each recording.

In timeseries plots, data are presented as mean values +/- SEM. In boxplots, the boxes extend from the Q1 to Q3, with the band inside the boxes representing the median. The whiskers extend to the smallest and largest values within 1.5 times the IQR. Source data are provided in 'Source Data1' file.

Supplementary Fig. 3 (related to Fig. 2)

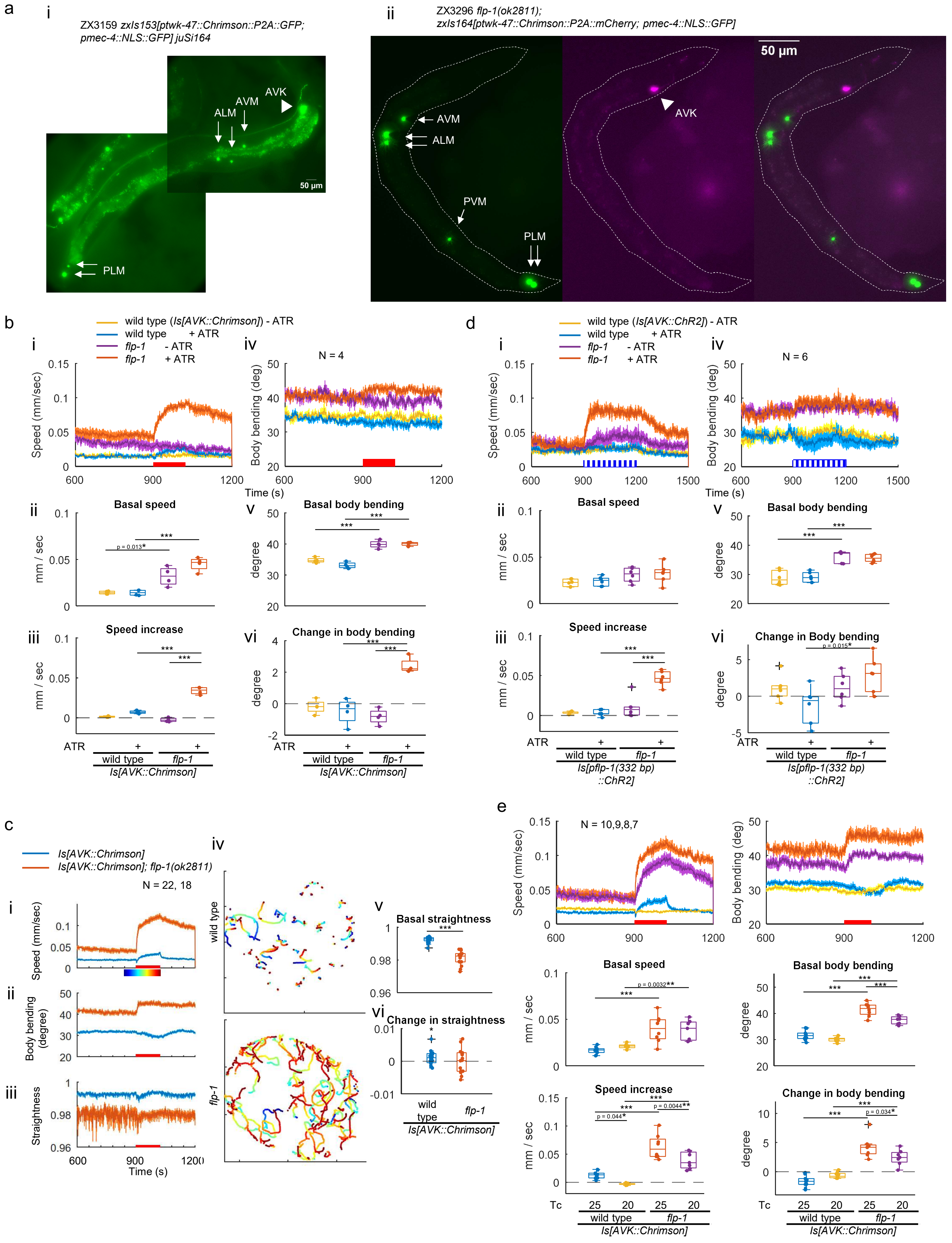

**Supplementary Figure 3. (related to Fig. 2)**

**a.** (i) Green fluorescence of *zxIs153[ptwk-47::Chrimson::P2A::GFP; pmec-4::NLS::GFP]* strain was imaged.
(ii) Green and red fluorescence of *flp-1(ok2811); zxIs164[ptwk-47::Chrimson::P2A::mCherry; pmec-4::NLS::GFP]* strain was imaged.

**b.** Wild type and *flp-1(ok2811)* mutant animals expressing Chrimson specifically in AVK were cultivated on NGM plates with or without supplementation of ATR for 3 days and subjected to behavioral analysis with MWT with continuous red light illumination. Tukey test was performed. ***p < 0.001. Approximately 30-100 animals were involved in each recording.

**c.** In addition to locomotion speed (i) and body bending (ii) for wild type (blue) and *flp-1* mutants (orange) straightness of locomotion direction (iii) was shown. Representative trajectories from 840 to 1020 sec are shown in iv, with color code representing time, as shown in i. Note that *flp-1* mutants tend to cluster at the edge of bacterial lawn[6]. (v) Basal straightness indicates mean value of t = 840~899. ***p < 0.001 (two-tailed Welch test). (iv) Change in straightness indicates mean values of t = 961-1020 subtracted by basal values. p = 0.0318* for wild type and 0.9899 for *flp-1* mutants (one sample t-test).

**d.** Wild type and *flp-1(ok2811)* mutant animals expressing ChR2 in AVK by the *flp-1(trc)* promoter were cultivated on NGM plates with or without supplementation of ATR for 3 days and subjected to behavioral analysis with MWT with pulsed blue light illumination (0.08 mW, 250 ms, 1 Hz, 300 times). Tukey test was performed. ***p < 0.001. Approximately 20-80 animals were involved in each recording.

**e.** Wild type and *flp-1(ok2811)* mutant animals expressing Chrimson specifically in AVK were cultivated on NGM plates at 20° C or 25° C and subjected to behavioral analysis with MWT. Measurements at 25° C are included in analyses in Fig. 2a-f and Supplementary Fig. 3c. Tukey test was performed. ***p < 0.001. Approximately 20-100 animals were involved in each recording.

In timeseries plots, data are presented as mean values +/- SEM. In boxplots, the boxes extend from the Q1 to Q3, with the band inside the boxes representing the median. The whiskers extend to the smallest and largest values within 1.5 times the IQR. Source data are provided in 'Source Data1' file.

Supplementary Fig. 4 (related to Fig. 3)

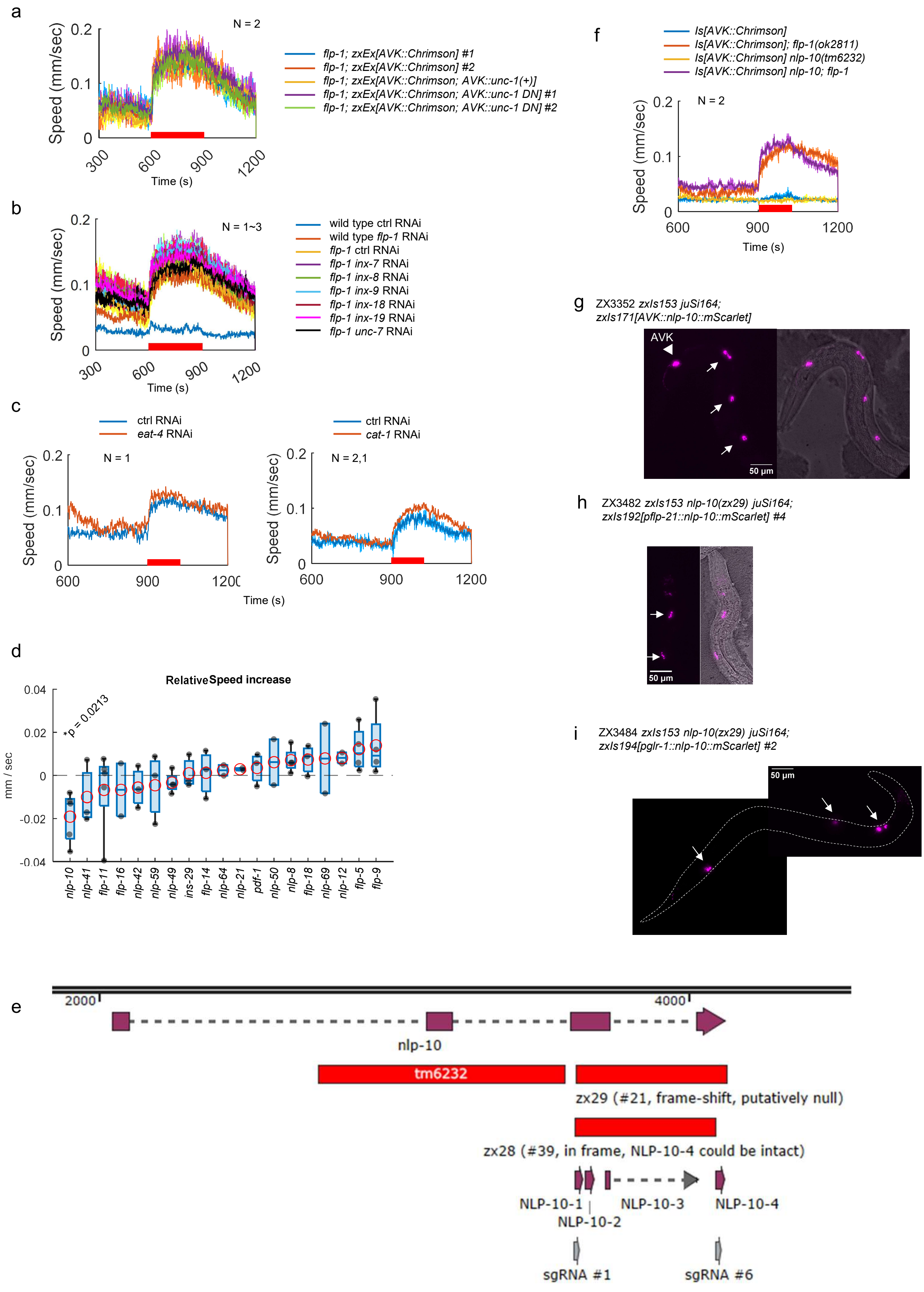

**Supplementary Figure 4. NLP-10 is responsible for speed increase following AVK-photoactivation (related to Fig. 3)**

**a.** *flp-1(ok2811)* mutant animals expressing Chrimson alone or in combination with either wild type or dominant negative form (N494C) of UNC-1 specifically in AVK were cultivated with ATR and subjected to behavioral analysis with MWT. Approximately 20-40 animals were involved in each recording.

**b.** Wild type and *flp-1(ok2811)* mutant of RNAi strains (*zxSi9[ptwk-47::rde-1:SL2:sid-1]*) expressing Chrimson in AVK were fed with HT115 bacteria expressing indicated dsRNA. Animals were then subjected to behavioral analysis with MWT. Approximately 40-100 animals were involved in each recording.

**c.** *flp-1(ok2811)* mutant of RNAi strain (*zxSi9[ptwk-47::rde-1:SL2:sid-1]; rde-1(ne300)*) expressing Chrimson in AVK were fed with HT115 bacteria expressing indicated dsRNA. Approximately 20-100 animals were involved in each recording.

**d.** *flp-1(ok2811)* mutant of RNAi strain (*zxSi9[ptwk-47::rde-1:SL2:sid-1]; rde-1(ne300)*) expressing Chrimson in AVK were fed with HT115 bacteria expressing dsRNA for indicated neuropeptides expressed in AVK.

Animals were subjected to behavioral analysis with red illumination. Relative speed increase was calculated by subtracting the speed increase of control animals analyzed on the same day from that of each species. Two-tailed t-test was performed. Source data are provided in 'Source Data1' file.

**e.** *nlp-10* genomic locus. Deletion sites in the respective mutant allele were indicated.

**f.** Animals expressing Chrimson in AVK with indicated genotypes were analyzed. Approximately 20-100 animals were involved in each recording.

**g-i.** Red fluorescence of indicated strains was imaged. The *twk-47* promoter was used for AVK-specific expression in (g). Bright field images were merged with fluorescence images on the right in (g) and (h). The outline of the animal was indicated by a dashed line in (i).

In timeseries plots, data are presented as mean values +/- SEM. In boxplots, the boxes extend from the Q1 to Q3, with the band inside the boxes representing the median. The whiskers extend to the smallest and largest values within 1.5 times the IQR.

Supplementary Fig. 5 (related to Fig. 3)

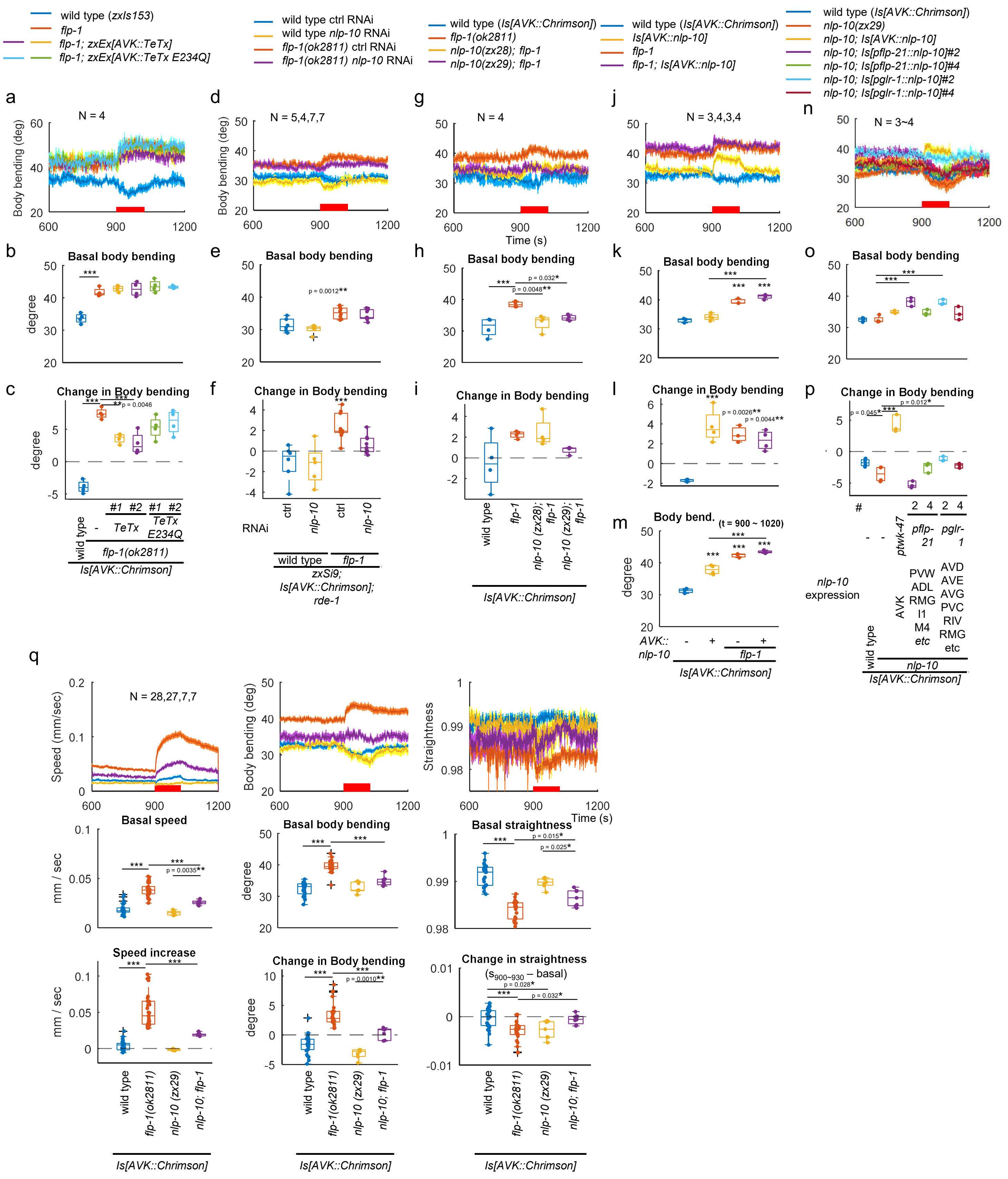

**Supplementary Figure 5. Body bending angle during experiments in Fig. 3**

**a-c.** Wild type and *flp-1(ok2811)* mutant animals, along with *flp-1* mutants expressing either wild type or enzymatically inactive (E234Q) TeTx in AVK, all expressing Chrimson in AVK, were cultivated with ATR overnight and subjected to behavioral analysis with MWT under red light illumination. (a) Body bending angle along time, (b) average body bending angle before illumination (t = 780 ~ 899, $b_o$) and (c) change in body bending angle during illumination (average body bending (t = 900 ~ 1020) – $b_0$) were plotted. Dunnett test was performed against *flp-1* mutants. ***$p < 0.001$.

**d-f.** Wild type and *flp-1(ok2811)* mutant RNAi strains expressing Chrimson in AVK were cultivated with HT115 bacteria carrying a control vector or a plasmid producing dsRNA for *nlp-10* in the presence of ATR for 3 days and subjected to behavioral analysis. Tukey test was performed.

**g-i.** Animals expressing Chrimson in AVK with indicated genotypes were analyzed. Tukey test was performed. ***$p < 0.001$.

**j-m.** Wild type and *flp-1(ok2811)* mutant animals expressing Chrimson with or without AVK-specific NLP-10 overexpression were analyzed. Tukey test was performed. ***$p < 0.001$.

**n-p.** Wild type and *nlp-10(zx29)* mutant animals, along with *nlp-10* mutants expressing NLP-10 in indicated neurons, all expressing Chrimson in AVK, were analyzed. Dunnett test was performed against *nlp-10* mutants. ***$p < 0.001$.

**q.** To analyze the straightness of animals of the indicated genotypes, datasets were extracted from experiments shown in Figs. 3 to 5. Tukey test was performed. ***$p < 0.001$.

In timeseries plots, data are presented as mean values +/- SEM. In boxplots, the boxes extend from the Q1 to Q3, with the band inside the boxes representing the median. The whiskers extend to the smallest and largest values within 1.5 times the IQR. Source data are provided in ‘Source Data1’ file.

Supplementary Fig. 6

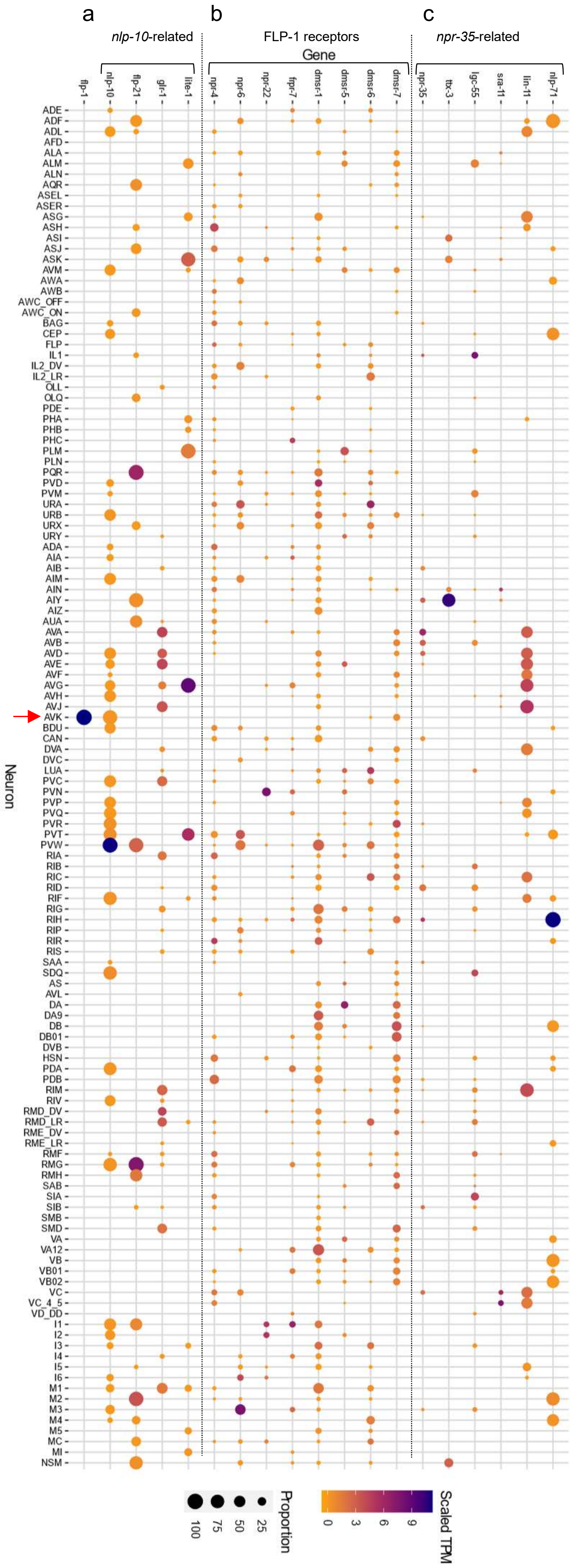


**Supplementary Figure 6. Expression pattern (related to Figs. 3-5)**

Heatmaps illustrating gene expression patterns were generated using CenGENApp[7] (https://cengen.shinyapps.io/CengenApp/). The heatmaps represent:

**a.** Genes associated with *nlp-10* rescue experiments.

**b.** FLP-1 receptors.

**c.** Genes whose promoters were utilized in *npr-35* rescue experiments.

Supplementary Fig. 7 (related to Fig. 4)

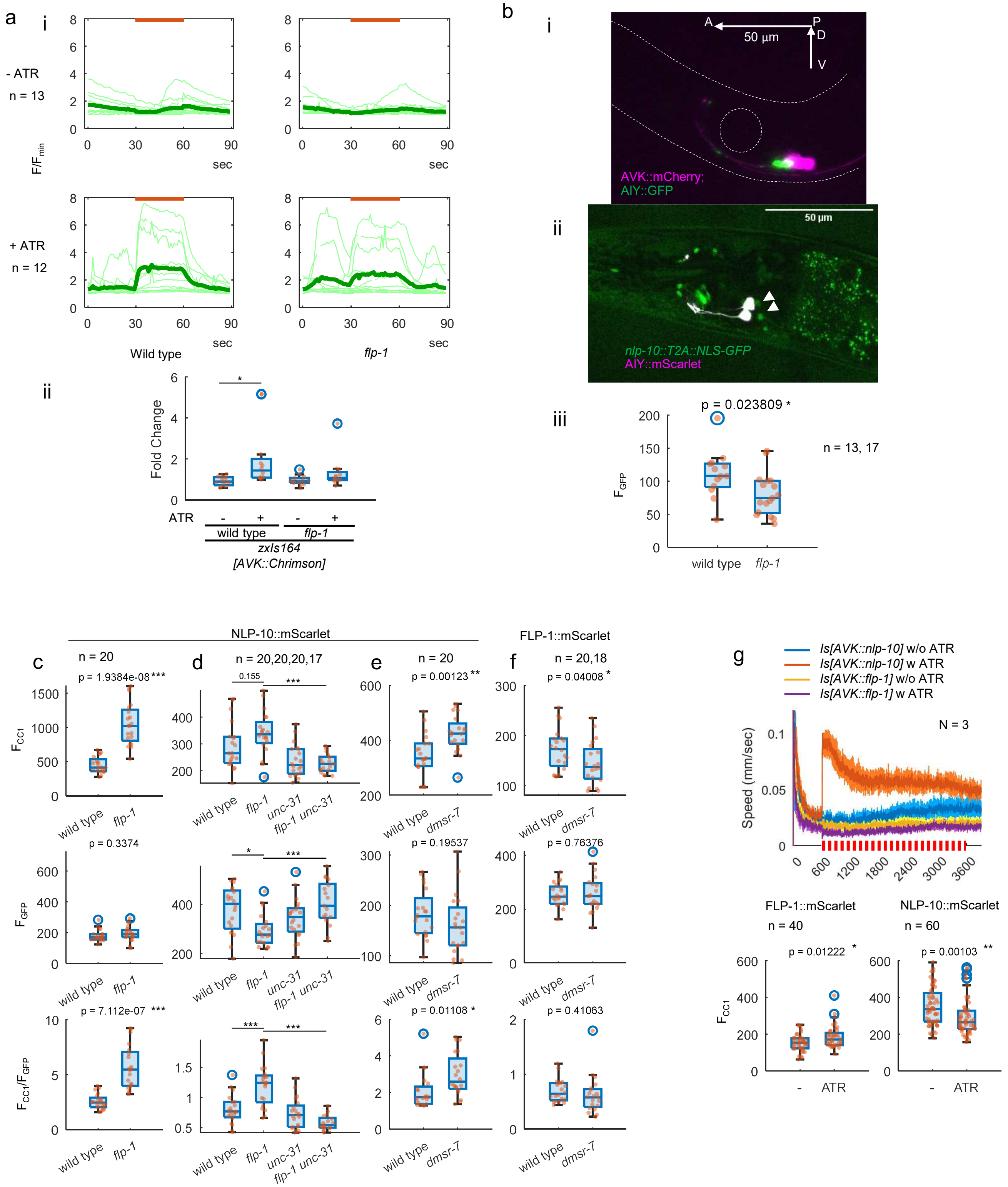

**Supplementary Figure 7. FLP-1 suppresses NLP-10 release from AVK (related to Fig. 4)**

**a.** Wild type and *flp-1(ok2811)* animals expressing GCaMP6s and Chrimson in AVK were cultivated with or without ATR and subjected to imaging at 1 frame per second (fps) with orange light (590 nm) illumination, as indicated. Raw images were subjected to background subtraction, and fluorescence intensities in AVK soma were quantified. (i) Normalized intensity was calculated by dividing raw intensity by the minimum value of each. (ii) Ratio of average of the normalized intensity before and during illumination was plotted. * indicates $p < 0.05$ (Tukey test).

**b. (i)** An animal expressing GFP in AIY and mCherry in AVK (*zxIs31[pflp-1(trc)::mCherry]; zxEx[pttx-3::GFP]*) was imaged by Zeiss Observer equipped with x40 objective lens and Kinetix22 camera. The animal outline and the position of terminal bulb were indicated by dashed lines. AVK is slightly posterior to AIY. (ii) An animal expressing NLS-GFP in *nlp-10*-expressing cells and mScarlet in AIY (*nlp-10(syb3179[nlp-10::T2A::3×NLS::GFP]) III; zxEx1523[pttx-3::mScarlet]*) was imaged with Leica SP8 confocal microscope with x63 objective lens. GFP and mScarlet were detected with HyD hybrid detector and normal PMT, respectively. Note that mScarlet fluorescence leaks to green channel. Arrowheads indicate AVK nuclei. (iii) Raw green images were subjected to background subtraction and fluorescence intensity (F) in AVK was quantified. Two-tailed Welch test was performed.

**c-f.** Fluorescence intensities of mScarlet in L4 larvae of animals of indicated genotype expressing NLP-10::mScarlet (c-e) or FLP-1::mScarlet (g) in AVK were measured in anterior coelomocytes ($F_{CC1}$). Fluorescence intensities of GFP expressed in AVK under the same promoter as NLP-10::mScarlet were also measured ($F_{GFP}$). These fluorescence intensities were plotted together with the ratio ($F_{CC1}$/ $F_{GFP}$). Two-tailed Welch test (c,e,f) and Tukey test (d) were performed. ***$p < 0.001$ (d).

**g.** Animals expressing NLP-10::mScarlet or FLP-1::mScarlet in AVK were cultivated with or without ATR and subjected to pulse illumination of 623 nm LED at 2 Hz (pulse length: 200 ms) for 1 hour. Locomotion speed was monitored with MWT. Approximately 40-120 animals were involved in each recording. Animals were then subjected to quantification of fluorescent intensities. $F_{CC1}$ in L4 larvae was plotted. Since GFP in these strains are not very bright, normalization was not performed. Two-tailed Welch test was performed.

In timeseries plots, data are presented as mean values +/- SEM. In boxplots, the boxes extend from the Q1 to Q3, with the band inside the boxes representing the median. The whiskers extend to the smallest and largest values within 1.5 times the IQR. Source data are provided in ‘Source Data2’ file.

# Supplementary Fig. 8 (related to Fig. 4)

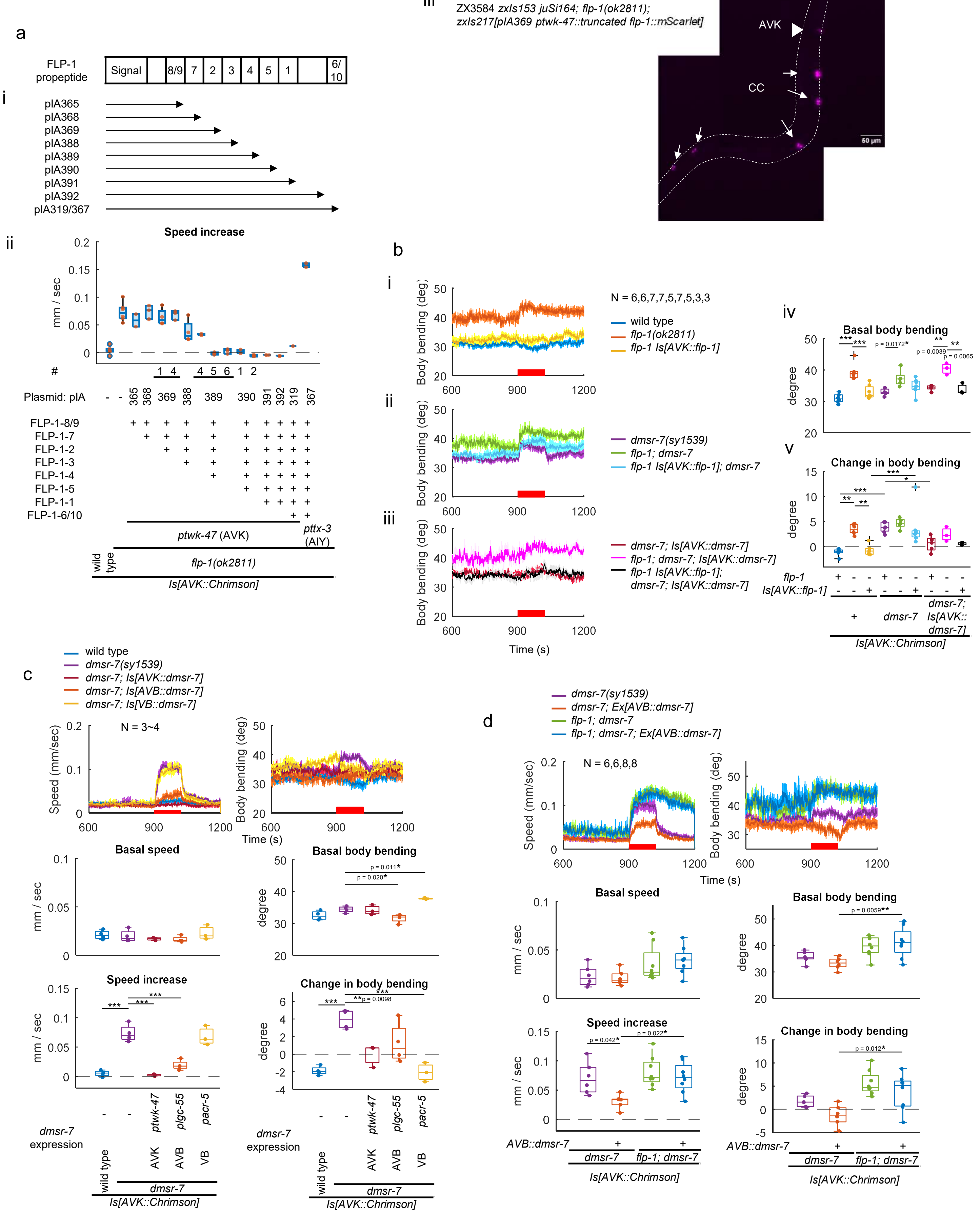

**Supplementary Figure 8. Autocrine feedback (related to Fig. 4)**

**a.** (i) The FLP-1 propeptide comprises multiple mature peptide species as numbered. A series of plasmids encoding truncated FLP-1 propeptide were generated for rescue experiments in (ii).

(ii) *flp-1* mutants expressing either full length or truncated FLP-1 propeptide along with wild type and *flp-1(ok2811)* mutant animals, all expressing Chrimson in AVK, were cultivated with ATR and subjected to behavioral analysis with MWT. (iii) Representative image of an animal expressing truncated FLP-1 propeptide containing FLP-1-8, 7 and 2 was shown. The animal outline was indicated by a dashed line. The arrowhead indicates AVK, and arrows indicate coelomocytes.

**b.** Animals of indicated genotypes expressing Chrimson in AVK were cultivated with ATR and subjected to behavioral analysis with MWT while red light was illuminated as indicated. (i-iii) Body bending angle along time, (iv) average body bending angle before illumination ($t = 780 \sim 899$, $b_o$) and (v) change in body bending angle during illumination (average body bending ($t = 900 \sim 1020$) – $b_0$) were plotted. Tukey test was performed. ***$p < 0.001$. Approximately 10-120 animals were involved in each recording.

**c.** Animals of indicated genotypes expressing Chrimson in AVK were cultivated with ATR and subjected to behavioral analysis with MWT while red light was illuminated as indicated. Dunnett test was performed against *dmsr-7* mutants. ***$p < 0.001$. Part of wild type, *dmsr-7* and *dmsr-7; Is[AVK::dmsr-7]* data are overlapping with Fig. 4b. Approximately 20-120 animals were involved in each recording.

**d.** Animals of indicated genotypes expressing Chrimson in AVK were picked and transferred to NGM plates with ATR, cultivated overnight and subjected to behavioral analysis with MWT while red light was illuminated as indicated. Tukey test was performed. Approximately 10-40 animals were involved in each recording.

In timeseries plots, data are presented as mean values +/- SEM. In boxplots, the boxes extend from the Q1 to Q3, with the band inside the boxes representing the median. The whiskers extend to the smallest and largest values within 1.5 times the IQR. Source data are provided in ‘Source Data2’ file.

Supplementary Fig. 9 (related to Fig. 5)

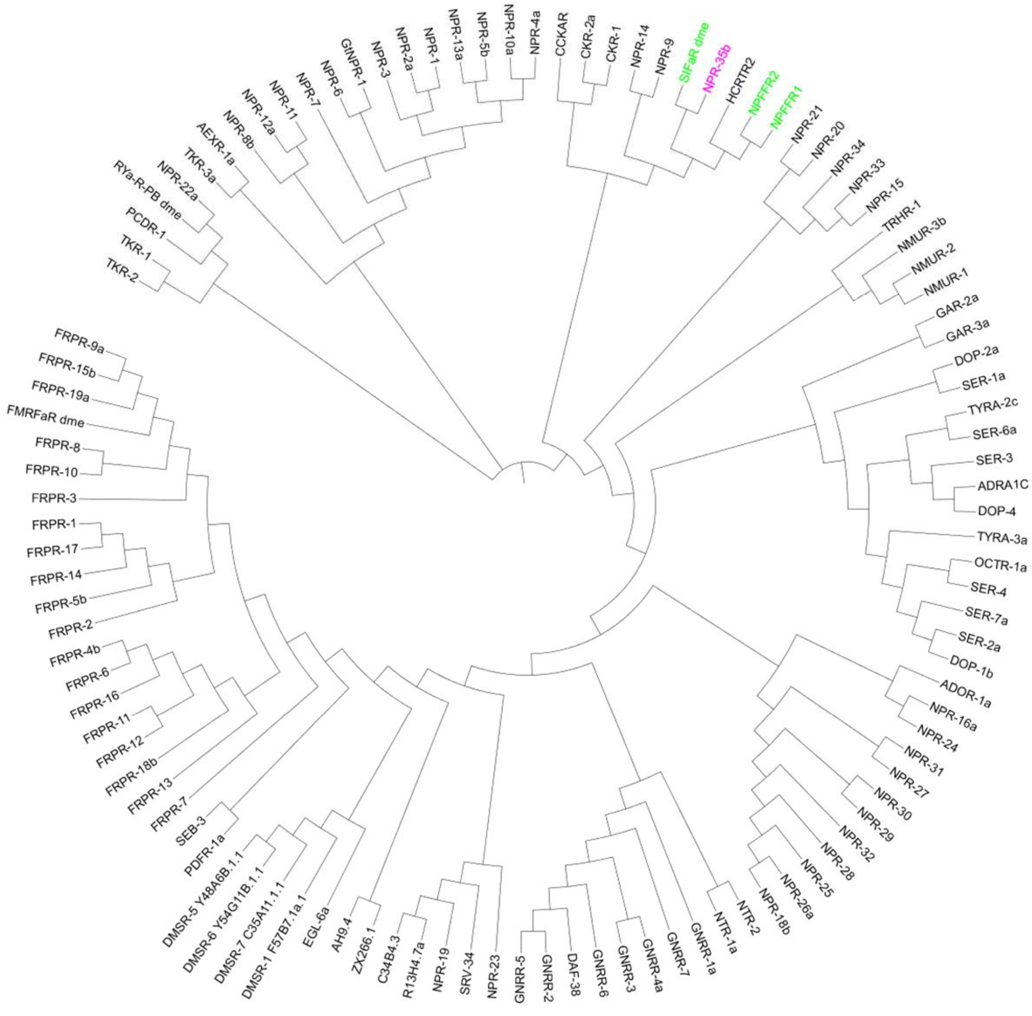


**Supplementary Figure 9. Phylogenetic tree of neuropeptide receptors in *C. elegans* and NPR-35 homologs (related to Fig. 5)**
Amino acid sequences of neuropeptide and monoamine receptors in *C. elegans,* along with homologs of NPR-35 in other species, were aligned using Clustal Omega. The resulting phylogenetic tree was generated and visualized with iTOL. NPR-35 and its homologs are highlighted in magenta and green, respectively.

Supplementary Fig. 10 (related to Fig. 5)

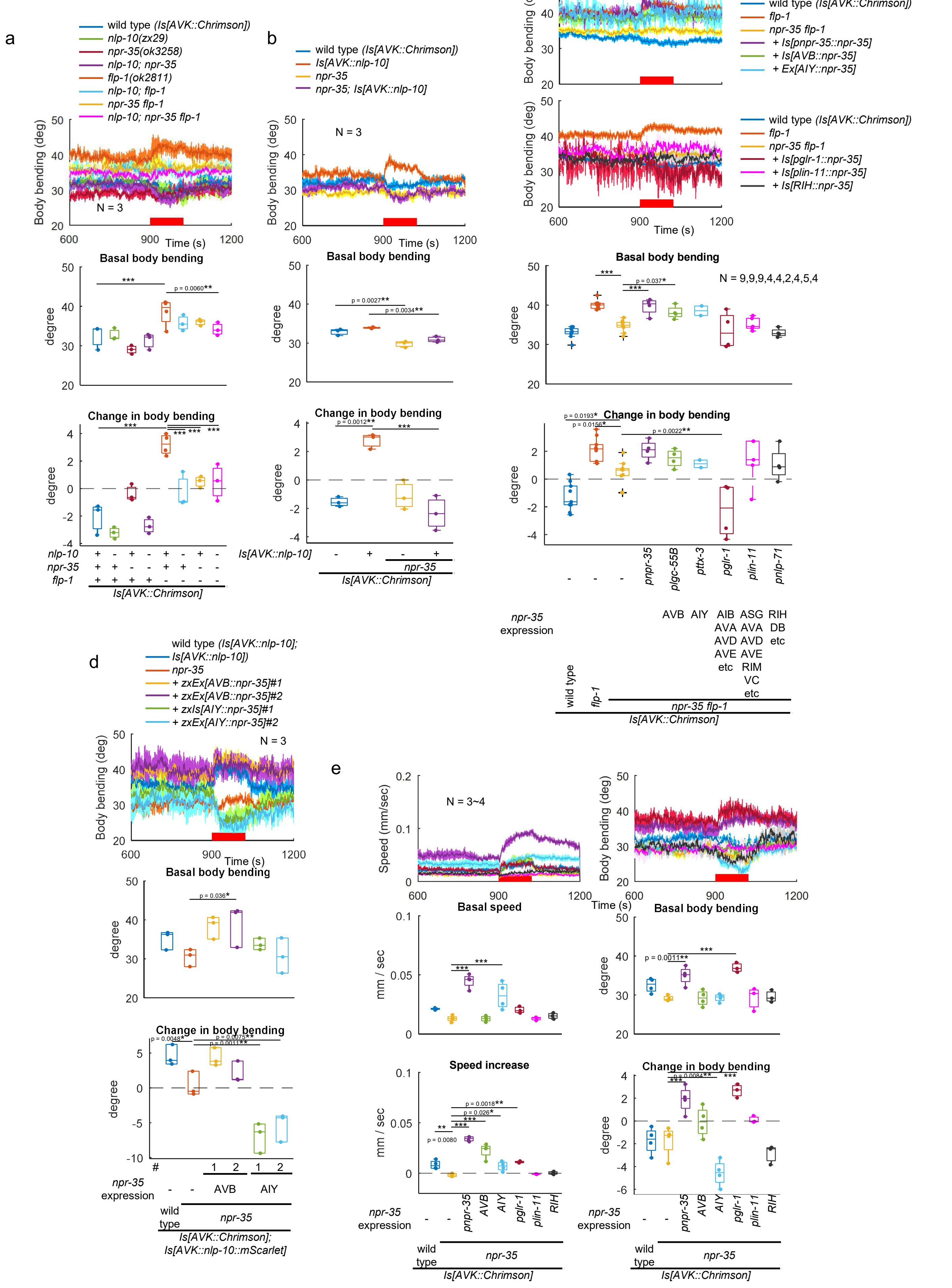

**Supplementary Figure 10. NPR-35 rescue (body bending related to Fig. 5)**

**a-d.** Measurements and analyses of body bending angle for animals in Fig. 5a-d are shown. Tukey test (a, b) or Dunnett test was performed against *npr-35 flp-1* double mutants (c) or *npr-35* mutants (d). ***p < 0.001.

**e.** Animals of indicated genotypes were analyzed with MWT. Dunnett test was performed against *npr-35* mutants. ***p < 0.001. Approximately 20-100 animals were involved in each recording.

In timeseries plots, data are presented as mean values +/- SEM. In boxplots, the boxes extend from the Q1 to Q3, with the band inside the boxes representing the median. The whiskers extend to the smallest and largest values within 1.5 times the IQR. Source data are provided in 'Source Data2' file.

Supplementary Fig. 11 (related to Fig. 6)

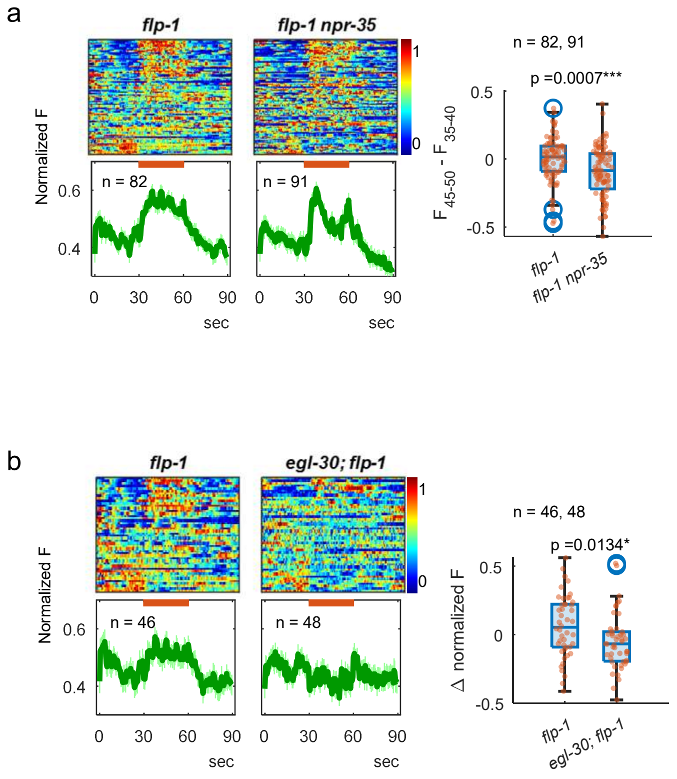


**Supplementary Figure 11. AIY Ca2+ imaging (related to Fig. 6)**

**a,b.** *flp-1(ok2811)* and *flp-1 npr-35* (a) or *egl-30; flp-1* (b) mutant animals expressing Chrimson in AVK and GCaMP6s in AIY were cultivated in the presence of ATR and subjected to imaging analysis. Images were acquired at 2 frames per second (fps) while orange light (590 nm) was illuminated as indicated. Raw images were subjected to background subtraction, and fluorescence intensity (F) in AIY varicosities were quantified. F was normalized between 0 to 1 by subtracting the minimum values followed by division by maximum values (after subtraction). Normalized F of individual recording were plotted in heat maps. Average of the normalized F was plotted with error bars indicating SEM.

a. Difference of normalized F between 45-50 sec and 35-40 sec was plotted.

b. Difference of normalized F before (21-30 sec) and after (31-40 sec) illumination was plotted. Data of *flp-1* mutants are partially overlapping with a.

Two-tailed Welch test was performed. In boxplots, the boxes extend from the Q1 to Q3, with the band inside the boxes representing the median. The whiskers extend to the smallest and largest values within 1.5 times the IQR. Source data are provided in 'Source Data2' file.

Supplementary Fig. 12 (related to Figs. 7-9)

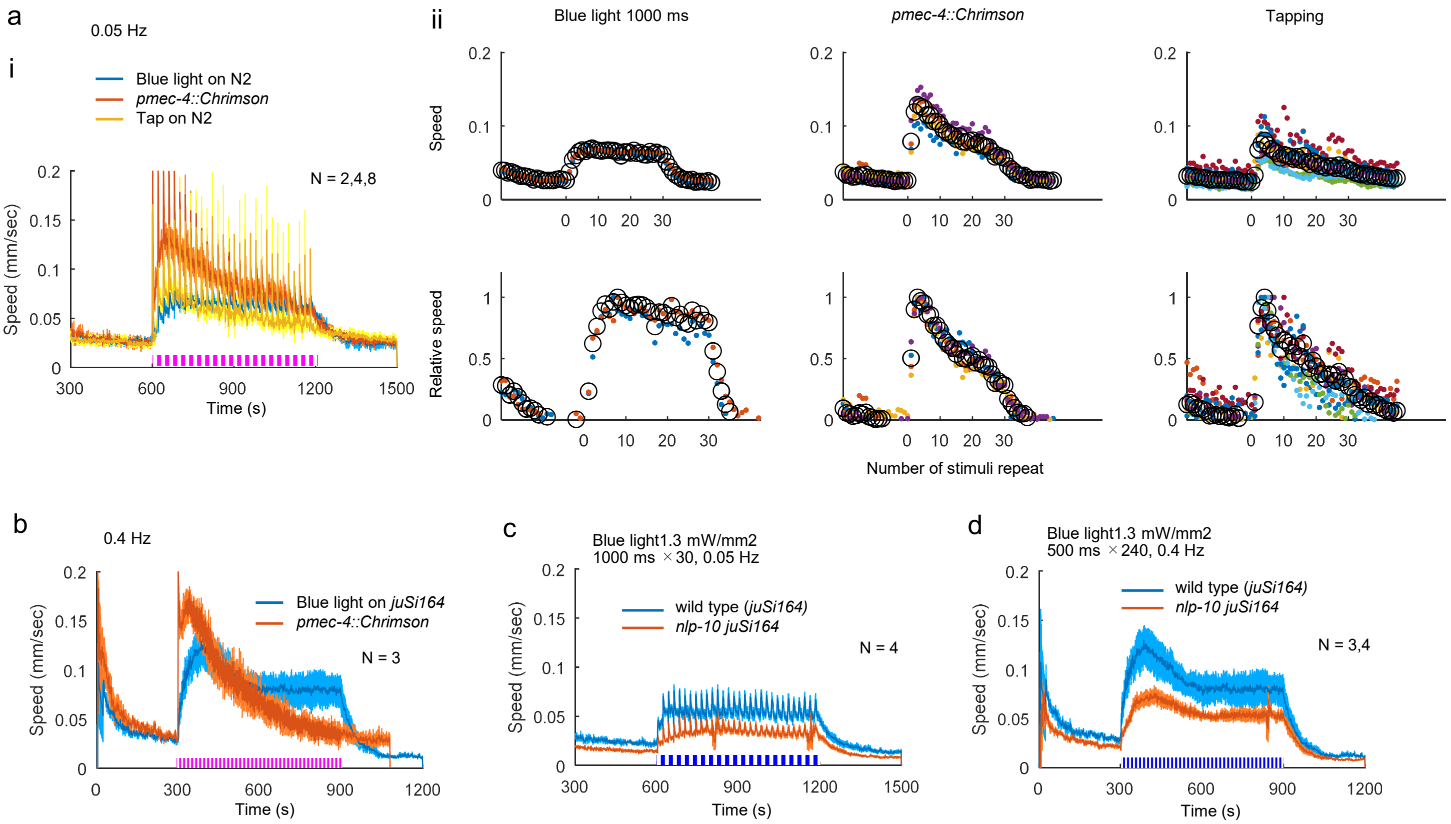


**Supplementary Figure 12. Response to repetitive stimuli (related to Figs. 7-9)**

**a.** (i) Wild type N2 strain was exposed to blue light (470 nm, 1.3 mW/mm2) for 1 sec each or tap-stimulated, and animals expressing Chrimson in tap-responsive neurons using *mec-4* promoter were exposed to red light for 200 ms each, 30 times at 0.05 Hz as indicated while their locomotion was monitored by MWT. Locomotion speed was plotted.

(ii) Mean speed between stimuli (top) and the mean speed between stimuli normalized between 0 and 1 (bottom) for individual recordings were dot-plotted with different colors. Black open circles indicate mean values among different recordings. Approximately 10-150 animals were involved in each recording. Source data are provided in 'Source Data2' file.

**b.** CZ20310 *juSi164* strain was exposed to blue light (470 nm, 1.3 mW/mm2) for 500 ms each, or animals expressing Chrimson in tap-responsive neurons were exposed to red light for 200 ms each, 240 times at 0.4 Hz as indicated while their locomotion was monitored by MWT. Locomotion speed was plotted. Approximately 40-150 animals were involved in each recording.

**c,d.** CZ20310 strain as wild type and *nlp-10* mutants, both carrying *juSi164,* were illuminated as in **b** or **c**. The trace of wild type in **e** is the replot of the one in **c**. Approximately 50-150 animals were involved in each recording.

In timeseries plots, data are presented as mean values +/- SEM.

## Supplementary References


1. Swierczek, N. A., Giles, A. C., Rankin, C. H. & Kerr, R. A. High-throughput behavioral analysis in C. elegans. *Nat. Methods* **8**, 592–8 (2011).
2. Jungreis, I. *et al.* Evidence of abundant stop codon readthrough in Drosophila and other metazoa. *Genome Res.* **21**, 2096–2113 (2011).
3. Prieto-Godino, L. L. *et al.* Olfactory receptor pseudo-pseudogenes. *Nature* **539**, 93–97 (2016).
4. Stiebler, A. C. *et al.* Ribosomal Readthrough at a Short UGA Stop Codon Context Triggers Dual Localization of Metabolic Enzymes in Fungi and Animals. *PLoS Genet.* **10**, e1004685 (2014).
5. Chew, Y. L., Grundy, L. J., Brown, A. E. X., Beets, I. & Schafer, W. R. Neuropeptides encoded by nlp-49 modulate locomotion, arousal and egg-laying behaviours in Caenorhabditis elegans via the receptor SEB-3. *Philos. Trans. R. Soc. B Biol. Sci.* **373**, 20170368 (2018).
6. Macosko, E. Z. *et al.* A hub-and-spoke circuit drives pheromone attraction and social behaviour in C. elegans. *Nature* **458**, 1171–5 (2009).
7. Taylor, S. R. *et al.* Molecular topography of an entire nervous system. *Cell* **184**, 4329-4347.e23 (2021).
