# Supplementary material for: Hierarchical regulation of functionally antagonistic neuropeptides expressed in a single neuron pair
Source: Nat Commun. 2024 Nov 3;15:9504. doi: 10.1038/s41467-024-53899-7 (PMC11532408; doi:10.1038/s41467-024-53899-7)
Supplement: Supplementary file 3 — Description of additional supplementary files [file 41467_2024_53899_MOESM3_ESM.pdf]

# Description of Additional Supplementary Files

**Supplementary Data 1 :** Lists of strains, plasmids, RNAi, primers and peptides used in this study are included.
