# Supplementary material for: Hierarchical regulation of functionally antagonistic neuropeptides expressed in a single neuron pair
Source: Nat Commun. 2024 Nov 3;15:9504. doi: 10.1038/s41467-024-53899-7 (PMC11532408; doi:10.1038/s41467-024-53899-7)
Supplement: Supplementary file 5 — Reporting Summary [file 41467_2024_53899_MOESM5_ESM.pdf]

nature portfolio

Corresponding author(s): Ichiro Aoki and Alexander Gottschalk

Last updated by author(s): Sep 24, 2024

Reporting Summary

Nature Portfolio wishes to improve the reproducibility of the work that we publish. This form provides structure for consistency and transparency in reporting. For further information on Nature Portfolio policies, see our Editorial Policies and the Editorial Policy Checklist.

Statistics

For all statistical analyses, confirm that the following items are present in the figure legend, table legend, main text, or Methods section.

n/a	Confirmed	
☐	☒	The exact sample size (n) for each experimental group/condition, given as a discrete number and unit of measurement
☐	☒	A statement on whether measurements were taken from distinct samples or whether the same sample was measured repeatedly
☐	☒	The statistical test(s) used AND whether they are one- or two-sided<br>*Only common tests should be described solely by name; describe more complex techniques in the Methods section.*
☒	☐	A description of all covariates tested
☐	☒	A description of any assumptions or corrections, such as tests of normality and adjustment for multiple comparisons
☐	☒	A full description of the statistical parameters including central tendency (e.g. means) or other basic estimates (e.g. regression coefficient) AND variation (e.g. standard deviation) or associated estimates of uncertainty (e.g. confidence intervals)
☐	☒	For null hypothesis testing, the test statistic (e.g. F, t, r) with confidence intervals, effect sizes, degrees of freedom and P value noted<br>*Give P values as exact values whenever suitable.*
☒	☐	For Bayesian analysis, information on the choice of priors and Markov chain Monte Carlo settings
☒	☐	For hierarchical and complex designs, identification of the appropriate level for tests and full reporting of outcomes
☒	☐	Estimates of effect sizes (e.g. Cohen's d, Pearson's r), indicating how they were calculated

Our web collection on statistics for biologists contains articles on many of the points above.

Software and code

Policy information about availability of computer code

Data collection	Multi-Worm Tracker, µManager 2.0, Arduino IDE
Data analysis	Multi-Worm Tracker (Choreography), MATLAB 2023a, ImageJ 1.54f

For manuscripts utilizing custom algorithms or software that are central to the research but not yet described in published literature, software must be made available to editors and reviewers. We strongly encourage code deposition in a community repository (e.g. GitHub). See the Nature Portfolio guidelines for submitting code & software for further information.

Data

Policy information about availability of data

All manuscripts must include a data availability statement. This statement should provide the following information, where applicable:

- Accession codes, unique identifiers, or web links for publicly available datasets
- A description of any restrictions on data availability
- For clinical datasets or third party data, please ensure that the statement adheres to our policy

Choreography output, image quantification data, and plasmid sequences, including their generation histories, are available in the figshare repository (https://doi.org/10.6084/m9.figshare.26513092). Due to their large size, the raw MWT outputs are available upon request. Source data are provided with this paper.

Research involving human participants, their data, or biological material

Policy information about studies with human participants or human data. See also policy information about sex, gender (identity/presentation), and sexual orientation and race, ethnicity and racism.

Reporting on sex and gender	All the experiments involving C. elegans were performed on hermaphrodites.
Reporting on race, ethnicity, or other socially relevant groupings	n/a
Population characteristics	n/a
Recruitment	n/a
Ethics oversight	n/a

Note that full information on the approval of the study protocol must also be provided in the manuscript.

Field-specific reporting

Please select the one below that is the best fit for your research. If you are not sure, read the appropriate sections before making your selection.

☒ Life sciences ☐ Behavioural & social sciences ☐ Ecological, evolutionary & environmental sciences

For a reference copy of the document with all sections, see nature.com/documents/nr-reporting-summary-flat.pdf

Life sciences study design

All studies must disclose on these points even when the disclosure is negative.

Sample size	No statistical methods were applied to predetermine sample size as the effect size was not known before the study. However, sample sizes reported here for the different experiments were matched to published experiments that used similar methodology, model systems and manipulations.
Data exclusions	No data were excluded during the analysis workflow.
Replication	For experiments involving C. elegans, measurements were performed on multiple independent animal generations. These measurements were then pooled for analysis as a single group. For experiments involving Xenopus laevis oocytes , all experiments were replicated multiple times in biologically independent samples. The precise replication numbers were reported in the manuscript.
Randomization	Animals for imaging and behavioral analyses and oocytes for electrophysiological analysis were randomly selected.
Blinding	Measurements were not blinded, since experiments were performed by a consistent workflow, and analyses included all the measurements and were automated and quantitative with no subjective thresholding.

Reporting for specific materials, systems and methods

We require information from authors about some types of materials, experimental systems and methods used in many studies. Here, indicate whether each material, system or method listed is relevant to your study. If you are not sure if a list item applies to your research, read the appropriate section before selecting a response.

Materials & experimental systems

n/a	Involved in the study	
☒	☐	Antibodies
☒	☐	Eukaryotic cell lines
☒	☐	Palaeontology and archaeology
☐	☒	Animals and other organisms
☒	☐	Clinical data
☒	☐	Dual use research of concern
☒	☐	Plants

Methods

n/a	Involved in the study	
☒	☐	ChIP-seq
☒	☐	Flow cytometry
☒	☐	MRI-based neuroimaging

Animals and other research organisms

Policy information about studies involving animals; ARRIVE guidelines recommended for reporting animal research, and Sex and Gender in Research

Laboratory animals	C. elegans strains used are listed in Supplementary Data.
Wild animals	This study did not involve wild animals.
Reporting on sex	All the experiments involving C. elegans were performed on hermaphrodites.
Field-collected samples	This study did not involve samples collected from the field.
Ethics oversight	Studies on C. elegans and Xenopus laevis oocytes do not require ethics oversight.

Note that full information on the approval of the study protocol must also be provided in the manuscript.

Plants

Seed stocks	n/a
Novel plant genotypes	n/a
Authentication	n/a
